# Overexpression of neuropilin-1 promotes constitutive MAPK signalling and chemoresistance in pancreatic cancer cells

**DOI:** 10.1038/sj.bjc.6602663

**Published:** 2005-06-14

**Authors:** J S Wey, M J Gray, F Fan, A Belcheva, M F McCarty, O Stoeltzing, R Somcio, W Liu, D B Evans, M Klagsbrun, G E Gallick, L M Ellis

**Affiliations:** 1 Department of Surgical Oncology, Unit 444, The University of Texas, MD Anderson Cancer Center, PO Box 301402, Houston, TX 77230-1402, USA; 2 Department of Cancer Biology, The University of Texas MD Anderson Cancer Center, Houston, TX, USA; 3 Department of Medical Oncology, The University of Texas MD Anderson Cancer Center, Houston, TX, USA; 4 Department of Pathology, Children's Hospital and Harvard Medical School, Boston, MA, USA

**Keywords:** chemotherapy, vascular endothelial growth factor receptor, anoikis, apoptosis

## Abstract

Neuropilin-1 (NRP-1) is a novel co-receptor for vascular endothelial growth factor (VEGF). Neuropilin-1 is expressed in pancreatic cancer, but not in nonmalignant pancreatic tissue. We hypothesised that NRP-1 expression by pancreatic cancer cells contributes to the malignant phenotype. To determine the role of NRP-1 in pancreatic cancer, NRP-1 was stably transfected into the human pancreatic cancer cell line FG. Signal transduction was assessed by Western blot analysis. Susceptibility to anoikis (detachment induced apoptosis) was evaluated by colony formation after growth in suspension. Chemosensitivity to gemcitabine or 5-fluorouracil (5-FU) was assessed by MTT assay in pancreatic cancer cells following NRP-1 overexpression or siRNA-induced downregulation of NRP-1. Differential expression of apoptosis-related genes was determined by gene array and further evaluated by Western blot analysis. Neuropilin-1 overexpression increased constitutive mitogen activated protein kinase (MAPK) signalling, possibly via an autocrine loop. Neuropilin-1 overexpression in FG cells enhanced anoikis resistance and increased survival of cells by >30% after exposure to clinically relevant levels of gemcitabine and 5-FU. In contrast, downregulation of NRP-1 expression in Panc-1 cells markedly increased chemosensitivity, inducing >50% more cell death at clinically relevant concentrations of gemcitabine. Neuropilin-1 overexpression also increased expression of the antiapoptotic regulator, MCL-1. Neuropilin-1 overexpression in pancreatic cancer cell lines is associated with (a) increased constitutive MAPK signalling, (b) inhibition of anoikis, and (c) chemoresistance. Targeting NRP-1 in pancreatic cancer cells may downregulate survival signalling pathways and increase sensitivity to chemotherapy.

Vascular endothelial growth factor (VEGF), one of the best-characterised proangiogenic factors, plays roles in both normal and pathologic angiogenesis. In particular, VEGF (VEGF-A, vascular permeability factor) overexpression has been associated with tumour progression and poor prognosis in a number of tumour systems (Dvorak *et al*, 1995; Takahashi *et al*, 1995; Fujimoto *et al*, 1998; Berns *et al*, 2003; Gorski *et al*, 2003). Vascular endothelial growth factor's effects are mediated primarily through two tyrosine kinase receptors: VEGFR-1 (Flt-1) and VEGFR-2 (Flk-1/KDR). More recently, two additional nontyrosine kinase receptors for the VEGF family have been identified, neuropilin-1 (NRP-1) and neuropilin-2, which are believed to function as co-receptors for VEGFR-1 and VEGFR-2 (Fuh *et al*, 2000; Whitaker *et al*, 2001). Although the VEGF receptors have been described as endothelial cell-specific, emerging evidence has documented their presence on tumour cells (Itakura *et al*, 2000; Masood *et al*, 2001). Vascular endothelial growth factor overexpression has been shown in a number of tumour systems (Fujimoto *et al*, 1998; Lee *et al*, 2000), where it is believed to function as a paracrine angiogenic factor affecting endothelial cell signalling; however, the presence of VEGF receptors on tumour cells raises the possibility of VEGF family members also acting in an autocrine fashion on the tumour itself.

In neurobiologic studies, the highly conserved glycoprotein NRP-1 was determined to mediate axonal guidance by forming complexes with plexins and propagating signals initiated by class 3 semaphorin ligands (Takahashi *et al*, 1999). Our laboratory and others have shown that NRP-1 is overexpressed in a variety of pancreatic tumour specimens and cell lines but not in nonmalignant pancreatic tissue (Parikh *et al*, 2003; Fukahi *et al*, 2004; Hansel *et al*, 2004). However, the functional significance of NRP-1 on tumour cells has not been elucidated.

We hypothesised that NRP-1 expression on pancreatic cancer cells contributes to the malignant phenotype. In this study, we overexpressed NRP-1 in a human pancreatic cell line (FG) with relatively low endogenous expression of NRP-1 and assessed the effects on downstream signalling pathways, detachment-induced apoptosis (anoikis), and chemoresistance. In a complementary experiment, we downregulated NRP-1 expression by small interfering RNA (siRNA) in a cell line with relatively high NRP-1 expression (Panc-1) and evaluated the effect of decreased NRP-1 expression on chemosensitivity. Neuropilin-1 overexpression in FG cells was found to increase constitutive mitogen-activated protein kinase (MAPK) signalling via both the extracellular signal-regulated kinase (ERK) and c-Jun NH_2_-terminal kinase (JNK) pathways, to enhance survival of cells grown in suspension, and to increase resistance to two chemotherapeutic agents. Furthermore, decreasing NRP-1 expression in Panc-1 cells enhanced their susceptibility to chemotherapy. Analysis of apoptosis-related gene expression by gene array also demonstrated that NRP-1 overexpression was associated with induction of the antiapoptotic regulator, MCL-1.

## MATERIALS AND METHODS

### Materials

Antibodies for Western blotting, immunoprecipitation (IP), and fluorescence-activated cell sorting (FACS) were as follows: polyclonal rabbit anti-phospho-ERK1/2 (p42/44) (Thr202/Tyr204; Cell Signaling Technology, Beverly, MA, USA), polyclonal rabbit anti-phospho-Akt (Ser473; Cell Signaling Technology), polyclonal rabbit anti-JNK (Thr183/Tyr185; Cell Signaling Technology), polyclonal rabbit anti-MAPK (Erk-1/2) (Oncogene Research Products, Cambridge, MA, USA), polyclonal rabbit anti-Akt (Cell Signaling Technology), monoclonal rabbit anti-JNK (56G8; Cell Signaling Technology), monoclonal mouse anti-NRP-1 (A-12; Santa Cruz Biotechnology, Santa Cruz, CA, USA), polyclonal rabbit anti-NRP-1 (H-286; Santa Cruz Biotechnology), polyclonal goat anti-SEMA3a (C-17) antibody (Santa Cruz Biotechnology), monoclonal mouse anti-vinculin (Sigma, St Louis, MO, USA), polyclonal rabbit anti-DFF45 (BD Biosciences, San Diego, CA, USA), monoclonal mouse anti-MCL-1 (22; Santa Cruz Biotechnology), fluorescein isothiocyanate (FITC)-conjugated goat anti-rabbit immunoglobulin G (IgG) (Jackson ImmunoResearch, West Grove, PA, USA), horseradish peroxidase (HRP)-conjugated sheep anti-mouse (Amersham Biosciences, Piscataway, NJ, USA), HRP-conjugated goat anti-rabbit (Bio-Rad Laboratories, Hercules, CA, USA), and HRP-conjugated rabbit anti-goat (Jackson ImmunoResearch). The chemotherapeutic agents gemcitabine (difluorodeoxycytidine) (Eli Lilly, Indianapolis, IN, USA) and 5-fluorouracil (5-FU) (50 mg ml^−1^ aqueous; American Pharmaceutical Partners, Schaumberg, IL, USA) were purchased from The University of Texas MD Anderson Cancer Center Pharmacy. Protein A/G agarose beads were purchased from Santa Cruz Biotechnology. Chemicals utilised were purchased from Sigma unless otherwise indicated.

### Cell lines and culture conditions

The FG cell line was originally established from COLO 357 human pancreatic cancer cells by Vezeridis *et al* (1990) and was kindly provided by IJ Fidler, PhD, DVM (The University of Texas MD Anderson Cancer Center, Houston, TX, USA). Panc-1 human pancreatic cancer cells were purchased from the American Type Culture Collection (Manassas, VA, USA). Cells were cultured and maintained in supplemented minimal essential medium (MEM) as previously described (Jung *et al*, 1999). Transfected cells were maintained in medium supplemented with 200 *μ*g ml^−1^ G418 (neomycin; Life Technologies). *In vitro* experiments were performed at 60–80% cell confluence, and cells were used at passages 3–15 after their receipt from the supplier or transfection.

### Stable transfection of NRP-1

The full-length human NRP-1 cDNA (Soker *et al*, 1998) was subcloned into the pcDNA3.1 vector (Invitrogen, Carlsbad, CA, USA) by standard techniques. Orientation was confirmed by restriction enzyme analysis and DNA sequencing. The resulting vector was stably transfected into human FG and Panc-1 pancreatic cancer cell lines using the FuGENE 6 transfection reagent (Roche Diagnostics Corporation, Indianapolis, IN, USA) according to the manufacturer's protocol. Control cells were transfected with the native pcDNA3.1 vector (mock transfectants). After 48 h, the medium was replaced by selective medium containing 400 *μ*g ml^−1^ G418. Clones were expanded, and successful transfection was confirmed by Western blot analysis using anti-NRP-1 antibodies (see below). Clones #1 and #9 expressed the highest levels of NRP-1 and were selected for further study.

### Creation of NRP-1 siRNA plasmids and cell lines

SiRNA expression plasmids were created using the pSilencer 1.0-U6 vector (Ambion, Austin, TX, USA) according to the manufacturer's directions. Neuropilin-1-specific target sequences were developed and designed using the Ambion siRNA web design tool. The two target sequences utilised were 5′-AAGCTCTGGGCATGGAATCAG-3′ and 5′-AAAGCCCCGGGTACCTTACAT-3′. Corresponding oligonucleotides with terminal Apa1 (5′) and R1 (3′) restriction sites were purchased from Invitrogen and ligated into the expression plasmid at compatible sites. Isolated clones were confirmed by DNA sequencing. Cell lines expressing decreased NRP-1 levels were created by transfecting Panc-1 cells with 0.5 ng of each of the siRNA plasmids and 10 ng of pcDNA G418-resistance promoter-less plasmid and selected in medium containing G418 as described above. Control cells were similarly transfected with scrambled NRP-1 target sequences and pcDNA plasmids. Neuropilin-1 expression levels in siRNA-transfected clones were determined by IP and Western blot analysis (see below). Clones #1 and #2 were shown to express <10% of baseline NRP-1 and were selected for further study.

### Fluorescence-activated cell sorting analysis of NRP-1-transfected cells

Cells were washed with phosphate-buffered saline (PBS) and detached with 0.1%. EDTA. Polyclonal rabbit anti-NRP-1 antibody (H-286) was added at 4 *μ*g million^−1^ cells, and cells were incubated on ice for 30 min. Cells were washed with cold PBS and incubated with FITC-conjugated goat anti-rabbit IgG at 7.5 *μ*g million^−1^ cells for 30 min on ice. Cells were washed, fixed in 1% paraformaldehyde, and analysed on an Epics XL-MCL flow cytometer (Beckman-Coulter, Miami, FL, USA). Positive staining (+) is designated as the % staining above background.

### Immunoprecipitation/Western blot hybridisation for NRP-1

Cells were lysed with RIPA-B protein lysis buffer (Parikh *et al*, 2003). Protein was quantitated by a commercially available modified Bradford assay (Bio-Rad protein assay, Bio-Rad Laboratories). For IP, 500 *μ*g of protein was preincubated for 1 h at 4°C with protein A/G agarose beads. Polyclonal rabbit anti-NRP-1 antibody (H-286) was then added at a 1 : 100 dilution and incubated at 4°C overnight with rotation. Immunoprecipitation samples were boiled with denaturing sample buffer for 5 min, and proteins were separated by sodium dodecyl sulphate–polyacrylamide gel electrophoresis (SDS–PAGE) on a 6% denaturing polyacrylamide gel and transferred onto a polyvinylidene difluoride (PVDF) membrane (Millipore Corp., Bedford, MA, USA) as previously described (Jung *et al*, 1999). Western blot samples utilised 50 *μ*g of protein from cell lysates, which were also boiled with denaturing sample buffer before electrophoresis. After blocking with 5% nonfat dry milk in Tris-buffered saline containing 0.1% Tween-20 (TBS-T), the membranes were probed with a monoclonal mouse anti-NRP-1 antibody (A-12) at a 1 : 200 dilution in 5% milk-TBS-T overnight at 4°C. The membranes were washed and incubated with an appropriate HRP-conjugated secondary antibody, and protein bands were visualised using a commercially available enhanced chemiluminescence (ECL) kit (Amersham Biosciences).

### Analysis of effect of NRP-1 transfection on signalling

Cells were grown to 60–70% cell confluence and incubated overnight in medium containing 5% FBS. Samples were prepared as described above. Activated signalling pathways were identified by incubation with polyclonal rabbit anti-phospho-ERK1/2, anti-phospho-JNK, or anti-phospho-Akt antibodies at a 1 : 1000 dilution in 5% milk-TBS-T. To verify accuracy of loading, membranes were stripped as previously described (Jung *et al*, 1999) and probed with anti-Erk-1/2, anti-JNK, or anti-Akt antibodies at a 1 : 1000 dilution in 5% milk-TBS-T. Detection of bands by ECL was performed using a secondary HRP-conjugated goat anti-rabbit antibody. Quantitative comparison of phosphorylated signalling intermediates was carried out by densitometric analysis of scanned images (NIH Image 1.63).

### Analysis of effect of conditioned medium from NRP-1-transfected cells on signalling

NRP-1-transfected or mock transfected FG cells were plated at 1 million cells/10 cm dish and incubated with 1% FBS-MEM for 48 h. Conditioned medium was then collected and centrifuged at 1000 r.p.m. for 5 min. Mock-transfected FG cells were plated at 60–70% confluence, and medium was changed to 1% FBS-MEM for overnight incubation. Mock-transfected cells were then treated for 5 or 15 min with conditioned medium from NRP-1 or mock transfectants. Cells were then lysed in RIPA-B lysis buffer, and Western blot analysis was performed as described above.

### Assessment of NRP-1 ligand production

Expression levels of the NRP-1 ligands VEGF and semaphorin3a (SEMA3a) by FG cells were investigated in order to assess for the possibility that NRP-1 overexpression could lead to induction of NRP-1 ligands, thus augmenting an autocrine loop. A human VEGF ELISA kit (R&D Systems, Minneapolis, MN, USA) was used to evaluate VEGF production in the conditioned medium and lysates from NRP-1 and mock transfected FG cells, according to the manufacturer's protocol. SEMA3a secretion was evaluated by Western blot of conditioned medium from transfected FG cells, utilising anti-SEMA3a antibody at a 1 : 200 dilution.

### Analysis of detachment-induced apoptosis (anoikis)

After NRP-1-transfected and mock-transfected FG cells were trypsinised, 1000 cells were either immediately plated in 10% MEM or grown in suspension for 48 h by rotation in a 15 ml Falcon tube with filter cap (BD Biosciences, Bedford, MA, USA) at 37°C in a standard incubator and then plated. Both suspended and non-suspended cells were allowed to grow for 1 week after plating. Colonies containing 10 or more cells were then counted on a grid, and the average of eight fields was calculated. Colony formation after growth in suspension was calculated as the percentage of such colonies formed relative to colonies formed by cells not subjected to growth in suspension.

### Analysis of effect of NRP-1 transfection on chemosensitivity

Transfected FG or Panc-1 cells (3–4 × 10^3^) were plated in 96-well plates. Following cell attachment after 24 h, medium was changed to 10% FBS-MEM with various concentrations of either gemcitabine or 5-FU, and cells were incubated for 24 or 48 h. Gemcitabine was reconstituted to 1 mM in PBS immediately prior to use. In order to quantitate surviving/proliferating cells, 0.5 mg ml^−1^ of 3-(4,5-dimethylthiazol-2-yl)-2,5-diphenyl-2H-tetrazolium bromide (MTT) was added and incubated for an additional 90 min. Medium and MTT were removed, dimethyl sulphoxide was added for 1 min to solubilise cells, and absorption was read at 570 nm in a spectrophotometer. In each experiment, cells were plated in quadruplicate and the average of the relative absorption (OD_570_) was used as an estimate of the number of metabolically active cells. Percentage of treated cells surviving compared to control cells not exposed to chemotherapy was calculated from the average OD_570_ values obtained in each experiment. Each experiment was performed at least in triplicate for statistical analysis. Analysis and graphing were performed utilising Sigmaplot 8.0 (SPSS Science Marketing Dept. Chicago, IL, USA).

### Analysis of apoptosis-related gene expression by SuperArray

Total RNA was extracted from subconfluent mock-transfected (pcDNA pool) and NRP-1-transfected (clone #1) FG cells using RNeasy Mini Spin Columns (QIAGEN, Valencia, CA) according to manufacturer's protocol. Biotin-labelled cDNA probes were prepared using the AmpoLabeling-LPR Kit (SuperArray, Frederick, MD, USA) and hybridised to the GEArray Q series Human Apoptosis Gene Array (SuperArray), according to the manufacturer's protocol. After chemiluminescent detection, data were analysed using ScanAlyze (www.rana.lbl.gov/EisenSoftware.htm; Eisen laboratory, Stanford University) and GEArray Analyzer software (SuperArray) in order to quantitate and normalise relative levels of gene expression. Protein expression of DFF45 and MCL-1 was assessed by Western blot analysis and densitometry as described above, utilising anti-DFF45 antibody (1 : 500 dilution) and anti-MCL-1 antibody (1 : 100 dilution) in 5% milk-TBS-T. Actin was used as a loading control.

## RESULTS

### NRP-1 overexpression by transfection in FG cells

**Figure 1:**
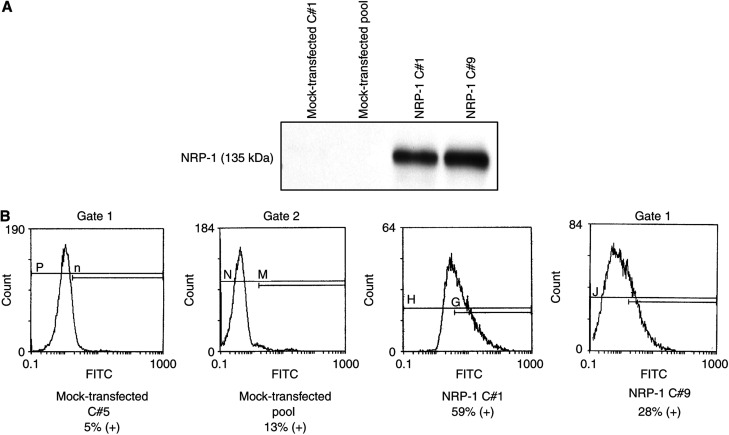
Effect of NRP-1 transfection on NRP-1 levels in FG cells. (**A**) Immunoprecipitation and Western blot analysis of lysates from mock-transfected (pool and C#1) and NRP-1-transfected clones (NRP-1 C#1 and C#9) confirmed overexpression of NRP-1 in transfectants. (**B**) FACS analysis using a polyclonal rabbit anti-NRP-1 antibody (H-286) demonstrated a two- to five-fold increase in NRP-1 expression in NRP-1-transfected cells. Data shown are representative of results from multiple experiments.

Given the observation that NRP-1 is expressed at higher levels in pancreatic tumours than in normal pancreatic tissue (Parikh *et al*, 2003), we first chose to study the effect of overexpression of NRP-1 in a cell line with relatively low endogenous expression, the FG human pancreatic carcinoma cell line. Immunoprecipitation/Western blot analysis demonstrated high levels of NRP-1 expression in NRP-1-transfected FG cells compared to parental or mock-transfected FG cells (Figure 1A). To confirm these findings, FACS analysis was also performed and confirmed a two- to five-fold increase in NRP-1 expression in transfected cells compared to control cells, based upon the percentage of cells expressing NRP-1 above background levels (Figure 1B). Similar results were confirmed after repeating experiments several times.

### Constitutive signalling in NRP-1-overexpressing FG cells

**Figure 2:**
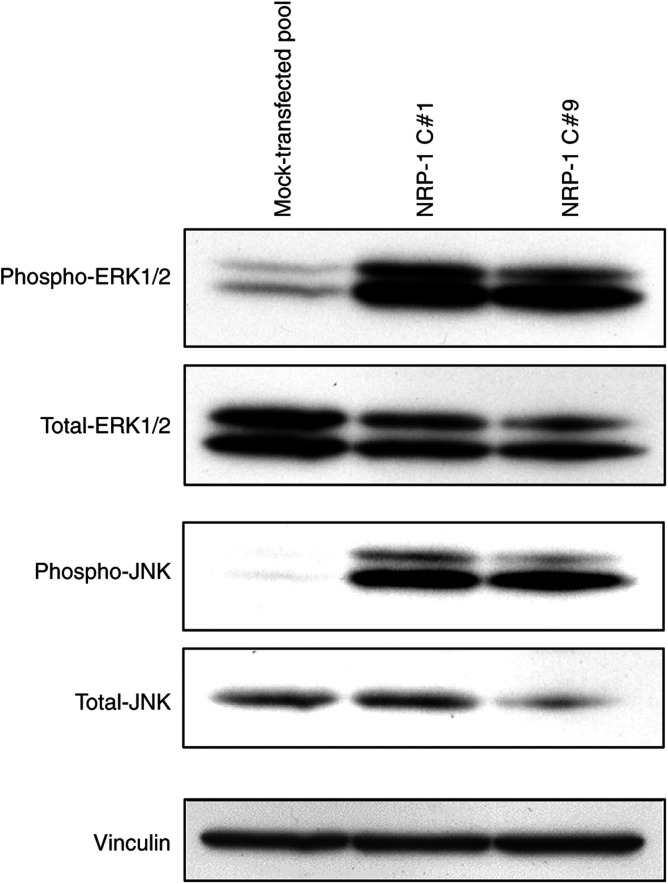
Effect of NRP-1 overexpression on signalling in pancreatic cancer cells. Neuropilin-1-transfected and mock-transfected FG cells were analysed by Western blotting for phosphorylated and total ERK1/2 and JNK. Both NRP-1-transfected clones (NRP-1 C#1 and C#9) demonstrated at least a six-fold increase in ERK1/2 and four-fold increase in JNK phosphorylation compared to mock transfectants. Total ERK1/2, total JNK, and vinculin are shown as loading controls.

The functionality of the overexpressed NRP-1 protein was then assessed for activation of downstream signalling intermediates. Compared to mock transfectants, NRP-1 transfectants demonstrated a >six-fold increase in constitutive ERK1/2 and >four-fold increase in constitutive JNK phosphorylation (Figure 2). In contrast, no differences were detected in phosphorylated Akt levels (data not shown). These findings were confirmed in repeat studies.

### Effect of conditioned medium from NRP-1-transfected cells on signalling

**Figure 3:**
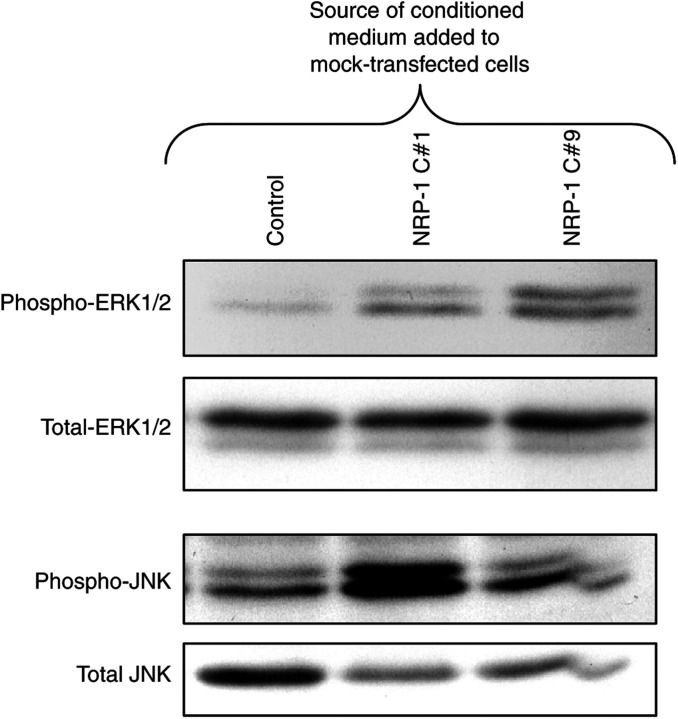
Effect of conditioned medium from NRP-1-transfected FG cells on signalling in mock-transfected FG cells. Mock-transfected (Control) or NRP-1-transfected FG cells were grown for 48 h in medium containing 1% FBS. Conditioned medium was collected and applied to mock-transfected FG cells. Mock-transfected control cells were left untreated. After incubation for 5 min (ERK) or 15 min (JNK), cells were lysed, and Western blot analysis for phosphorylated ERK1/2 or JNK was performed. Treatment with conditioned medium from NRP-1-transfected cells (NRP-1 C#1 and C#9) led to moderate upregulation in ERK1/2 phosphorylation and at least three-fold upregulation of JNK phosphorylation relative to cells treated with conditioned medium from mock-transfectants. Control-treated cells had relatively unchanged signalling compared to untreated cells (data not shown).

Since NRP-1 overexpression resulted in such a consistent increase in ERK1/2 and JNK signalling, we sought to determine whether this was caused by induction of an autocrine pathway. Conditioned medium from mock-transfected or NRP-1-transfected FG cells was collected and applied to mock-transfected FG cells, and the effect on signalling intermediates was assessed. Nearly a two-fold induction of ERK1/2 phosphorylation was consistently detected in cells exposed to conditioned medium from NRP-1-overexpressing cells, and a greater than three-fold increase was seen in JNK phosphorylation, similar to levels seen in NRP-1-overexpressing cells themselves (Figure 3). Low levels of phosphorylated ERK1/2 and JNK were seen in control cells grown in 1% medium (data not shown). These findings suggest that the upregulated MAPK signalling observed in NRP-1-overexpressing cells is due to an autocrine pathway regulated by a secreted factor.

Since VEGF-A is a major ligand for NRP-1, VEGF-A levels were evaluated in cell supernatants and lysates by enzyme-linked immunosorbent assay. Although VEGF-A levels were slightly higher in lysates from NRP-1-transfected FG cells than in mock transfectants, the level of secreted VEGF-A in conditioned medium from NRP-1 transfectants was approximately 25% lower than in conditioned medium from mock-transfected controls (data not shown), suggesting that VEGF-A is unlikely to be solely responsible for the observed signalling alterations. Expression of semaphorin-3a (SEMA3a), the natural ligand for NRP-1 in the nervous system, was also assessed in NRP-1-transfected FG cells by Western blot analysis, and its levels were unchanged by NRP-1 overexpression (data not shown). Thus, NRP-1 overexpression appears to induce MAPK signalling, at least in part via an autocrine pathway, although the mediator of this does not appear to be solely dependent on VEGF or SEMA3a.

### Effect of NRP-1 overexpression on susceptibility to anoikis

**Figure 4:**
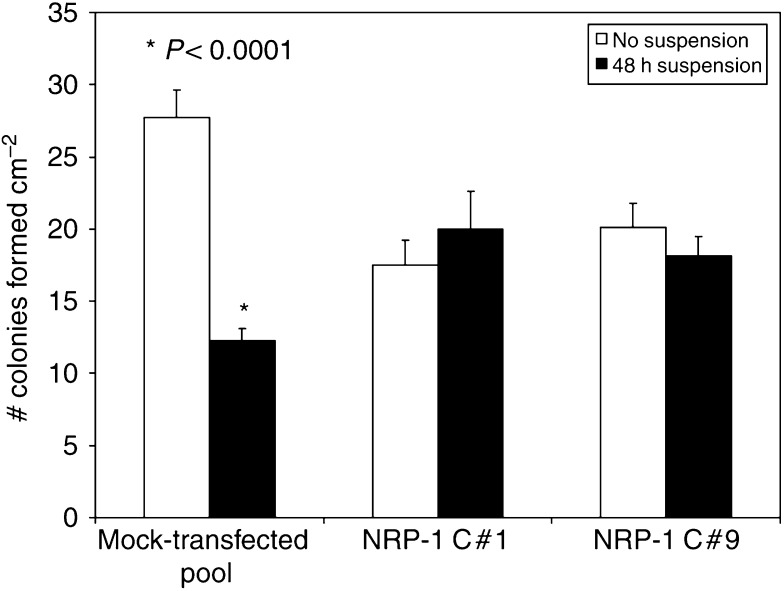
Increased resistance of NRP-1-transfected FG cells to anoikis. Neuropilin-1-overexpressing cells (NRP-1 C#1 and C#9) or mock transfectants were plated on 10-cm dishes or grown in suspension for 48 h prior to plating. Cells were allowed to grow for 1 week, and colonies of more than 10 cells each were counted on a grid. Data are expressed as mean colony number per cm^2^; error bars represent s.e.m. Control cells formed nearly 50% fewer colonies per cm^2^ compared to NRP-1-overexpressing cells (*P*<0.0001). The colony-forming ability of NRP-1-overexpressing cells was unchanged by growth in suspension. Experiments were performed at least in duplicate; graph shown is representative of findings.

Since the MAPK/ERK pathway has been associated with increased survival of cells and anoikis resistance in some systems (Fukazawa *et al*, 2002), we were interested in determining how its induction affects the ability of FG cells to survive particular stressors. Normal epithelial cells subjected to loss of substrate adherence undergo apoptosis in a process known as anoikis (Frisch and Francis, 1994). This apoptotic response is often lost in cancer cells (Swan *et al*, 2003), and this loss may facilitate their ability to survive detachment and to implant elsewhere as a metastasis. Control FG cells and NRP-1-overexpressing FG cells were plated either after standard substrate-adherent growth or after 48 h of incubation in suspension. Survival was assayed by colony formation after 1 week of adherent growth. In contrast to mock-transfected cells, which only formed about half as many colonies per cm^2^ after growth in suspension, both NRP-1-overexpressing clones formed equal numbers of colonies, with or without being subjected to growth in suspension (*P*<0.0001) (Figure 4). These findings suggest that NRP-1 overexpression provides cells with a survival advantage when they lose substrate attachment.

### Effect of NRP-1 overexpression on chemosensitivity of FG cells

**Figure 5:**
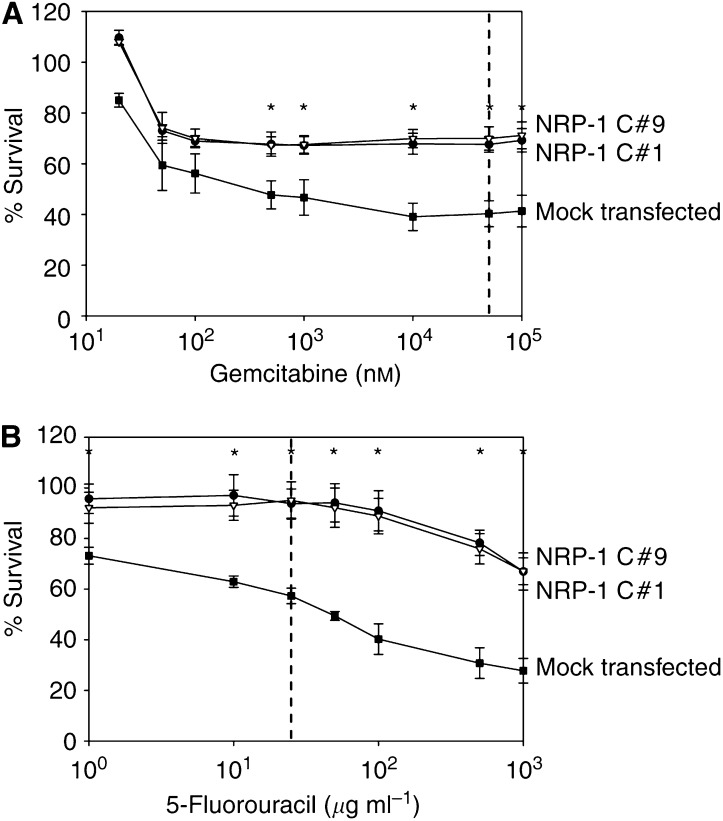
Promotion of chemoresistance by NRP-1. (**A**) NRP-1-transfected or mock-transfected FG cells were plated on a 96-well plate and treated with various doses of gemcitabine. After incubation for 24 h, cell survival was assessed by MTT assay. At concentrations of gemcitabine similar to peak plasma levels after bolus chemotherapy (50 000 nM, indicated by dashed line), NRP-1-overexpressing cells demonstrated greater chemoresistance (higher survival rate) compared to controls (*P*⩽0.005). Filled squares, mock-transfected pool; filled circles, NRP-1 clone #1 (C#1); open triangles, NRP-1 clone #9 (C#9); ^*^P<0.05. (**B**) NRP-1-transfected or mock-transfected FG cells were plated on a 96-well plate and treated with various doses of 5-FU. After incubation for 24 h, cell survival was assessed by MTT assay. At concentrations of 5-FU similar to peak plasma levels after bolus chemotherapy (25 *μ*g ml^−1^, indicated by dashed line), NRP-1-overexpressing cells demonstrated greater chemoresistance (higher survival rate) compared to controls (*P*<0.005). Similar results were seen at 48 h (not shown). Filled squares, mock-transfected pool; filled circles, NRP-1 clone #1 (C#1); open triangles, NRP-1 clone #9 (C#9); ^*^P<0.05.

NRP-1 overexpression enhanced the resistance of FG cells to anoikis; therefore, we hypothesised that NRP-1 overexpression also promoted survival of cells exposed to other stressors. The role of NRP-1 expression in mediating chemoresistance was of particular interest, since most chemotherapeutic agents have limited efficacy in patients with pancreatic cancer. We focused our studies on the effects of gemcitabine on pancreatic cancer cells *in vitro*, as this agent is approved for clinical use by the US Food and Drug Administration. After 24 h of gemcitabine exposure, MTT assay demonstrated improved survival in both NRP-1-overexpressing clones relative to the mock transfectants at doses around 100 nM, near the 50% inhibitory concentration (IC_50_) of gemcitabine in FG cells (*P*<0.03) (Figure 5A). An IC_50_ dose was not achieved in the NRP-1 transfected cells at this time point, even at supratherapeutic doses. The survival advantage was even more dramatic (*P*⩽0.005) at 50 000 nM, a therapeutically relevant concentration within the range of the peak plasma levels achieved following bolus gemcitabine chemotherapy (De Pas *et al*, 2000; Fogli *et al*, 2002). At this dose, at least 30% more of the NRP-1-transfected cells survived compared to control cells. At 48 h, the majority of all cells were nonviable, and no statistically significant difference between NRP-1-transfected and mock-transfected cells was observed (data not shown).

To verify that this chemoresistance was not a phenomenon unique to gemcitabine, studies were repeated using 5-FU, a different chemotherapeutic agent often used in treatment of pancreatic cancer. Again at 24 h, NRP-1 overexpression afforded cells a greater than 40% improvement in survival at doses near the IC_50_ (25 *μ*g ml^−1^) (*P*<0.005) (Figure 5B), which also approximates the peak plasma levels of 5-FU achieved during chemotherapy (Bocci *et al*, 2000). The statistically significant survival difference persisted at 48 h after exposure to this agent (data not shown).

### Effect of NRP-1 knockdown on chemosensitivity in Panc-1 cells

**Figure 6:**
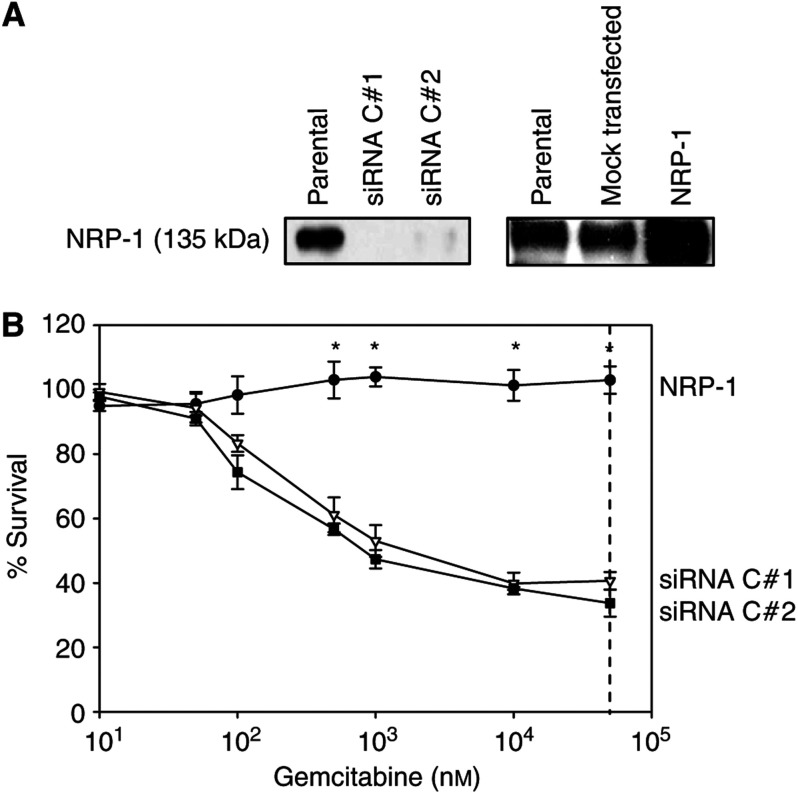
Association of NRP-1 levels with chemoresistance to gemcitabine in Panc-1 transfectants. (**A**) Panc-1 cells were transfected with full-length NRP-1, siRNA directed against NRP-1, or mock-transfected control vectors (pcDNA). Selected clones were screened for NRP-1 expression by Western blot analysis using either a polyclonal or monoclonal anti-NRP-1 antibody. Relative levels of NRP-1 expression were compared by densitometry. Neuropilin-1-transfected clones with at least two-fold overexpression were selected for further study. Selected clones (C#1 and #2) with functional siRNA demonstrated a decrease in NRP-1 expression by >90% and were selected for further study. Mock-transfected cells demonstrated levels of NRP-1 expression similar to those in parental cells. (**B**) NRP-1-transfected, mock-transfected, and siRNA-transfected (NRP-1-depleted) Panc-1 cells were plated on a 96-well plate and treated with various doses of gemcitabine. After incubation for 24 h, cell survival was assessed by MTT assay. At concentrations of gemcitabine similar to peak plasma levels after bolus chemotherapy (50 000 nM, indicated by dashed line), siRNA-transfected cells with low levels of NRP-1 (C#1 and C#2) were significantly more susceptible to chemotherapy compared to NRP-1-overexpressing cells (*P*⩽0.001). Mock-transfected control cells demonstrated chemoresistance that varied (not significantly) by experiment but remained at levels between those of NRP-1-overexpressing and NRP-1-depleted siRNA-transfected cells (data not shown). Similar effects were seen at 48 h (not shown). Filled circles, NRP-1 transfectant; open triangles, siRNA transfectant C#1; filled squares, siRNA transfectant C#2; ^*^P<0.006.

Having demonstrated that NRP-1 overexpression provides a survival advantage for FG cells exposed to two chemotherapeutic agents, we sought to determine whether decreased NRP-1 expression would render pancreatic cancer cells more susceptible to these drugs. Since FG cells express relatively low levels of NRP-1 at baseline, we utilised the Panc-1 cell line, a human pancreatic cancer cell line with endogenously high levels of NRP-1 to develop clones with downregulated NRP-1 expression, utilising siRNA technology. Western blot analysis and densitometry indicated that siRNA-transfected clones expressed NRP-1 protein at less than 10% of endogenous levels (Figure 6A). Using stable transfection of NRP-1, we also developed clones with at least two-fold higher levels of NRP-1 than parental or mock transfectants.

Both the NRP-1-overexpressing and siRNA clones were then assayed for chemosensitivity to gemcitabine and 5-FU by MTT assay. SiRNA-transfected Panc-1 cells proved to be much more susceptible to gemcitabine at both 24 and 48 h compared to NRP-1-overexpressing clones, which demonstrated almost no sensitivity to chemotherapy. At the therapeutically relevant dose of 50 000 nM, the difference in survival following gemcitabine treatment between NRP-1-overexpressing cells and cells with decreased NRP-1 expression was >50% and highly significant (*P*<0.001) (Figure 6B). In fact, even at supratherapeutic levels of gemcitabine, the IC_50_ dose was not achieved in the NRP-1 transfectants. Similarly, NRP-1 downregulation by siRNA rendered the Panc-1 cells more susceptible to treatment by 5-FU compared to NRP-1 transfectants (*P*<0.05) at the clinically relevant dose of 25 *μ*g ml^−1^ (data not shown). The susceptibility of parental Panc-1 cells varied from experiment to experiment but remained intermediate relative to that of either the NRP-1-overexpressing or knockout clones (data not shown).

### Effect of NRP-1 transfection on apoptosis-related gene expression

Since the previous experiments demonstrated an association between NRP-1 and apoptosis resistance, we hypothesised that NRP-1 expression might induce changes in genes associated with regulation of apoptosis. Comparison of gene expression between FG-NRP-1 transfectants and mock transfectants by a commercial apoptosis-related gene microarray kit and associated analysis software demonstrated more than 25 genes with a greater than two-fold change in expression level (data not shown); however, the most striking NRP-1-induced changes were noted in two antiapoptotic genes: DNA fragmentation factor 45/alpha (DFF45), which exhibited a four-fold increase, and a Bcl-2 homologue, myeloid cell leukemia sequence 1 (MCL-1), which exhibited a three-fold increase in gene expression level.

**Figure 7:**
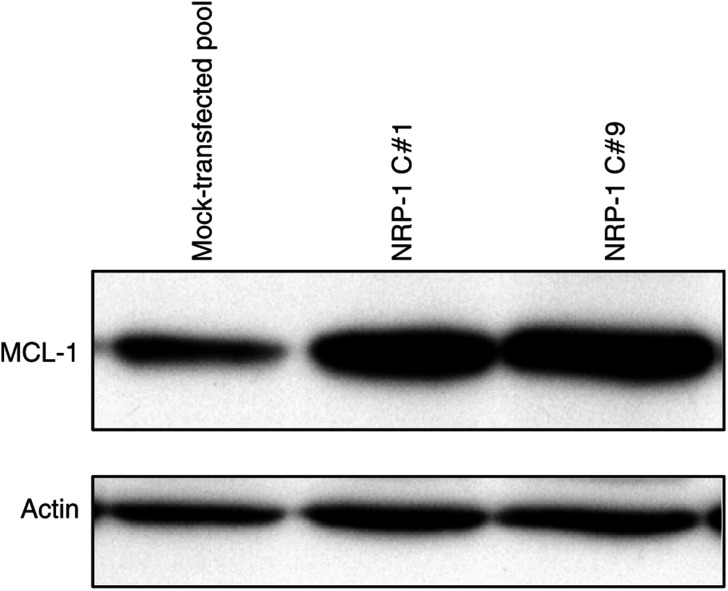
Effect of NRP-1 overexpression on the antiapoptotic protein, MCL-1. Analysis of apoptosis-related gene expression by gene microarray revealed a three-fold increase in gene expression of the antiapoptotic Bcl-2 homologue, *MCL-1*. In order to confirm upregulation of the MCL-1 protein, Western blot analysis of the FG mock transfectants and NRP-1 transfectants was performed. A nearly two-fold increase in MCL-1 protein expression was observed in the NRP-1-overexpressing cells. Actin levels are shown as a loading control.

In order to confirm these findings, Western blot analysis of the FG-NRP-1 and mock-transfected cells was carried out to assess relative protein expression levels of DFF45 and MCL-1. While DFF45 protein levels were relatively similar in NRP-1 overexpressing cells and controls (data not shown), MCL-1 protein levels were nearly two-fold higher in NRP-1-transfected cells (Figure 7). The reproducibility of MCL-1 induction by NRP-1 overexpression and the significance of this gene on survival/chemoresistance in other cell lines have yet to be determined.

## DISCUSSION

The importance of NRP-1 as a functional receptor in neurobiology is well established, but its role in tumour biology has only recently been recognised. This study provides evidence that altering the level of NRP-1 in pancreatic cancer cell lines causes functional changes relevant to tumour cell survival. Overexpression of NRP-1 in the FG human pancreatic cell line led to induction of both the ERK1/2 and JNK signalling pathways, possibly in an autocrine fashion, as the conditioned medium from NRP-1-overexpressing cells also increased phosphorylated-ERK1/2 and JNK in control cells. We also demonstrated that NRP-1 overexpression in the FG cell line enhanced cell survival following growth in suspension and exposure to the chemotherapeutic agents, gemcitabine and 5-FU. Correspondingly, downregulating NRP-1 levels by siRNA in the Panc-1 cell line led to increased susceptibility to gemcitabine relative to cells overexpressing the protein. Preliminary data by microarray suggest that NRP-1 may lead to induction of antiapoptotic genes, including MCL-1.

Our interest in NRP-1 originated from earlier studies demonstrating its function as a co-receptor for VEGFR-2, facilitating VEGFR-2 binding of VEGF_165_ in endothelial and enhancing cell migration (Soker *et al*, 1998). In a prostate cancer model, transfection of NRP-1 increased VEGF_165_ binding, basal cell motility, and tumour size *in vivo* (Miao *et al*, 2000), suggesting that the receptor functions in a VEGF-dependent fashion to enhance tumorigenicity. However, the survival effects demonstrated in our study occurred in two cell lines that do not express VEGFR-2, although they do express VEGFR-1. While NRP-1/VEGFR-1 interactions may occur, the addition of a VEGFR-1 blocking antibody did not enhance the chemosensitivity of NRP-1-transfected or mock-transfected cells (data not shown), suggesting that VEGFR-1 is not essential for this chemoresistance phenomenon. Moreover, the use of soluble VEGFR-1 and bevacizumab (humanised anti-VEGF antibody; Genentech, South San Francisco, CA, USA) had only a minor effect on cell signalling and survival. Indeed, more recent studies suggest that NRP-1 functions in tumour cells other than as a VEGFR co-receptor. One such study demonstrated that VEGF acted as an autocrine survival factor for NRP-1-expressing breast cancer cells, protecting them from hypoxia-induced apoptosis in the absence of VEGFR-2 expression through activation of the phosphatidylinositol 3′-kinase pathway (Bachelder *et al*, 2001). Neuropilin-1 has also been implicated in chemotaxis of breast cancer cell lines through autocrine pathways involving both VEGF and SEMA3a by complexing with plexin-A1 (Bachelder *et al*, 2003). Interestingly, whereas the short 40 amino-acid intracellular domain had previously been thought to be incapable of independent signalling, one study utilising chimeric NRP-1 suggests that the intracellular domain of NRP-1 alone may mediate VEGF-dependent migration in endothelial cells (Wang *et al*, 2003). Thus, the mechanisms by which NRP-1 induces functional changes in tumour cells require further elucidation.

In addition to a VEGFR-independent function of NRP-1, our studies also implicate a novel ligand for NRP-1, other than the traditional ligands, VEGF and SEMA3a. Our conditioned medium studies show that a soluble factor produced by NRP-1 overexpressing cells activates MAPK signalling in control cells, although neither of the traditional NRP-1 ligands, VEGF-A and SEMA3a, were shown to be upregulated in NRP-1 transfected cells (data not shown). Moreover, addition of recombinant VEGF_165_ or SEMA3a did not induce a significant increase in phosphorylated ERK1/2 and JNK in mock-transfected or parental cells, nor did they alter the chemoresistance of the NRP-1-transfected or mock-transfected cells in our study (data not shown). Thus, the possibility of a novel VEGF-independent and semaphorin-independent NRP-1 function remains. This is of particular interest, since the soluble factors secreted by NRP-1 expressing cells may enable them to influence the survival of adjacent cells without NRP-1 overexpression in a paracrine fashion. The identity of this factor remains to be determined.

The mechanisms by which NRP-1 induces chemoresistance may be multifactorial. Although the increased proliferative rate of cancer cells is one reason for their susceptibility to chemotherapy, this alone is unlikely to have caused the significant differences in chemosensitivity observed between NRP-1-transfected and mock-transfected cells, since NRP-1 transfection caused only a slight decrease in proliferation relative to mock transfection (<30%; data not shown). Recent studies have demonstrated induction of signalling pathways specifically associated with gemcitabine resistance in pancreatic carcinoma. Nuclear factor-*κ*B (NF-*κ*B) is one such factor whose constitutive activation has been associated with gemcitabine resistance in pancreatic carcinoma cell lines (Arlt *et al*, 2003). In our studies, activation of NF-*κ*B (as shown by nuclear translocation) did not occur, as NRP-1-transfected and mock-transfected cells showed identical patterns of NF-*κ*B nuclear and cytoplasmic localisation (data not shown). Others studies have shown that phosphorylation of the Src nonreceptor tyrosine kinase is associated with inherent or acquired gemcitabine resistance in pancreatic cancer cells (Duxbury *et al*, 2004); however, we detected no differences in phosphorylated Src between NRP-1-transfected and mock-transfected FG cells (data not shown). Thus, our observation of NRP-1-mediated chemoresistance is likely to occur via an alternative pathway yet to be determined.

Chemoresistance may, in fact, be one of several consequences of induction of MAPK signalling cascades. Our studies showed increased constitutive ERK1/2 and JNK signalling as a consequence of NRP-1 overexpression. Activation of ERK (Fukazawa *et al*, 2002; Subauste *et al*, 2004) or JNK signalling (Yang *et al*, 2003; Curtin and Cotter, 2004; Yu *et al*, 2004) in association with apoptosis resistance/cell survival is well documented in the literature in a variety of model systems. Moreover, at least one study has demonstrated that inhibition of ERK signalling in pancreatic cancer cell lines leads to downregulation of Bcl-2 and other antiapoptotic proteins, including MCL-1 (Boucher *et al*, 2000). Thus, NRP-1-induced activation of these MAPK signalling cascades may promote survival by induction of downstream apoptotic regulators, including MCL-1, as observed in our studies. Interestingly, at least one other study has noted that altered regulation of apoptosis-regulating genes including MCL-1 appears to be associated with acquired chemoresistance of pancreatic cancer cells (Shi *et al*, 2002). While our studies of NRP-1 and MCL-1 have only yielded very preliminary data in one cell line, the possibility of pathways linking NRP-1, signal transduction, antiapoptotic proteins, and cell survival are intriguing. Further identification of apoptotic regulators whose gene expression is altered by NRP-1 transfection may be warranted.

In summary, this study demonstrates that overexpression of NRP-1 in a pancreatic cancer cell line increased MAPK signalling through both the ERK and JNK pathways in an autocrine fashion. These pathways appear to promote survival of the pancreatic cancer cell line, specifically against anoikis and chemotherapy-induced apoptosis, possibly through induction of anti-apoptotic regulators. Although results of *in vitro* assays cannot be directly extrapolated to clinical response owing to the complexity of tumour biology in humans, our findings may have therapeutic implications. Since anoikis resistance may correlate with the ability of cells to survive detachment from the primary tumour mass and proliferate after migration to a distant site, targeting NRP-1 may increase anoikis in tumour cells and thereby decrease formation of metastases. Furthermore, the possibility of targeting therapies to decrease the level or function of NRP-1 in NRP-1-overexpressing tumours may lead to enhanced chemosensitivity in a previously chemoresistant tumour. In addition, the association between NRP-1 levels and these apoptosis-resistance characteristics may enable NRP-1 to be used as a prognostic marker. The elucidation of the role of NRP-1 in tumour biology remains at an early stage, but the use of NRP-1 as a prognostic factor and/or therapeutic target holds promise.
